# Geospatial heterogeneity of hotspots for incidence and late-stage diagnosis of breast, colorectal, and lung cancer

**DOI:** 10.21203/rs.3.rs-7330140/v1

**Published:** 2025-08-22

**Authors:** Andrew P. Loehrer, Heather A. Carlos, Julia E. Weiss, Chelsea V. Leversedge, Julia E. Katter, Joseph D. Phillips, Dana Ferrari-Light, Tracy Onega, Xun Shi

**Affiliations:** Dartmouth-Hitchcock Medical Center; Geisel School of Medicine at Dartmouth; Geisel School of Medicine at Dartmouth; Geisel School of Medicine at Dartmouth; Geisel School of Medicine at Dartmouth; Dartmouth-Hitchcock Medical Center; Dartmouth-Hitchcock Medical Center; University of Utah, Huntsman Cancer Institute; Dartmouth College

**Keywords:** Hotspot, Cancer Incidence, Access to Care, Cancer Control, Prevention

## Abstract

**Purpose:**

Cancer control relies on the identification of populations at risk (hotspots) of new or late-stage cancer diagnoses. However, the extent to which hotspots differ between cancer sites or between outcome measures has been poorly characterized. We sought to determine the geospatial heterogeneity of hotspots of breast, colorectal, and lung cancer incidence and late-stage diagnoses.

**Methods:**

We identified adult patients diagnosed with female breast, colorectal, and lung cancer between 2010 and 2019 in Indiana. To identify hotspots for incidence and late-stage diagnoses, we disaggregated the patient residential location information from the Census block group level to the approximated individual point level. Statistically significant hotspots were identified with kernel ratio estimation. Total areas of hotspots and overlap between hotspots were compared.

**Results:**

117,305 patients diagnosed with breast (n=51,623), colorectal (n=25,160), and lung (n=37,522) cancer were included. Geospatial visualization demonstrated marked spatial deviation, with little overlapping area between incidence and late-stage hotspots for all three cancer sites (32km^2^ – 165km^2^). However, there was greater overlap in late-stage hotspots between the different cancer sites, with total overlapping hotspot areas ranging from 408km^2^ – 1046km^2^.

**Conclusions:**

Our results demonstrate considerable geospatial heterogeneity of hotspots between different outcome measures and different sites of cancer. However, there are greater overlapping areas of late-stage hotspots, especially for breast and lung cancer. The use of disaggregated spatial data enables more granular, precise comparison of cancer hotspots. Greater overlap between late-stage breast and lung cancer suggests similar spatial drivers and the potential for coordinated cancer control interventions.

## INTRODUCTION

Cancer prevention and control efforts are contingent on the ability to identify populations and communities at greatest risk of cancer development and delayed, late-stage disease at time of diagnosis [1–3]. The utilization of aspatial data has repeatedly demonstrated specific population attributes associated with adverse cancer outcomes, including lower socioeconomic status, higher concentration or segregation of minority communities, and residence in more rural areas of the country [4–6]. While constructive in understanding general risks of adverse outcomes, these demographic characteristics alone fail to differentiate which specific communities are associated with such outcomes [7, 8]. Geospatial visualization, which incorporates spatial data, is critical in identifying specific communities experiencing worse than expected outcomes compared to surrounding areas.

Prior work has demonstrated areal clusters of hotspots for breast, colorectal, and lung cancer diagnoses and presentation with late-stage disease [9–11]. Additionally, many federal, state, and other professional cancer programs regularly evaluate cancer outcomes using various geospatial methodologies with larger areal units of evaluation, such as ZIP codes or counties [12–14]. However, significantly less is known about how to identify smaller areas of specific risk influence both the incidence of cancer and access to timely diagnosis. Furthermore, knowledge gaps remain regarding the similarity and overlap of hotspots between different cancer outcome measures or different cancer types. Consequently, policy or care delivery changes aimed at cancer prevention and control may fail to locate the appropriate communities to target or outcome measures to evaluate select interventions. Alternative approaches, such as using underlying population density to disaggregate spatial data, have been proposed for more precise and stable estimates of disease hotspots [15–17]. However, their ability to discriminate between hot spots of different outcome measures or between different cancer sites remains unclear.

The primary objective of this study is to evaluate the geospatial heterogeneity of hotspots of incidence and late-stage diagnoses of breast, colorectal, and lung cancer. Factors associated with higher incidence do not necessarily align with risk factors for presenting with later-stage cancer at the time of diagnosis, especially for breast cancer. Concurrently, the local factors contributing to hotspots of later-stage cancer may not overlap for different cancer sites. We hypothesize that hotspots will vary between sites of cancer (breast, colorectal, or lung) as well as outcome measures (incidence and late-stage diagnosis). Given more closely related drivers of timely access to care, we hypothesize that there will be greater overlapping areas of late-stage hotspots between the different cancer sites.

## MATERIALS & METHODS

### Cohort Development

**Figure 1:**
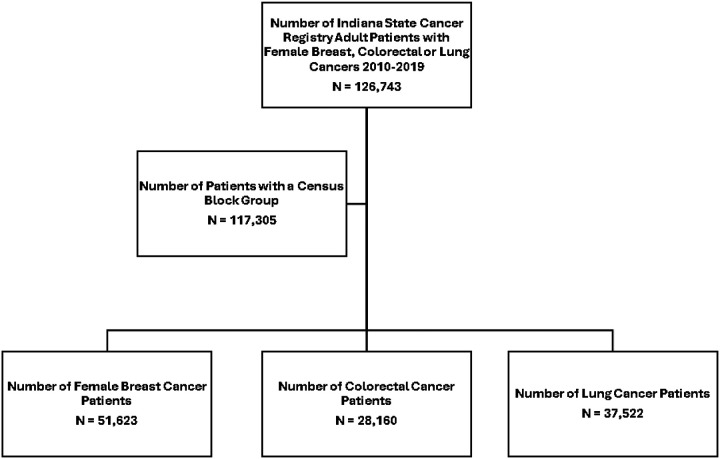
Cohort development from the Indiana State Cancer Registry (ISCR), STROBE Chart

Using data from the Indiana State Cancer Registry (ISCR), we identified all female patients 18 years or older diagnosed with breast (female only), colorectal, and lung cancer from 1/1/2010 through 12/31/2019 (Fig. 1). The ISCR captures nearly 100% of all new cancer diagnoses each year and is maintained in accordance with national standards set by the North American Association of Central Cancer Registries. International Classification of Disease – Oncology site and pathology codes were used to identify the cancer cases. Patients were excluded if they had missing residential location at the level of the United States Census block group. This study was deemed exempt from review by the Dartmouth-Hitchcock Institutional Review Board.

### Primary Outcomes

Our primary outcomes were incidence of breast, colorectal, or lung cancer and presentation with late-stage cancer at the time of diagnosis. Late-stage cancer diagnosis was determined for each cancer site using the American Joint Committee on Cancer (AJCC) 7th and 8th Edition staging system variables as captured in the ISCR [18]. A hierarchical calculation using both ISCR pathology and clinical staging data within the two time periods 2010–2017 and 2018–2019 defined stage [19]. This staging algorithm used pathology staging first and if pathology was absent, then clinical staging. An indicator variable for late-stage cancer diagnosis differentiated individuals classified as stage III or IV from those classified as stage 0, I, or II.

### Patient Characteristics

Patient demographic and clinical characteristics were used to describe our cohort using the statistical software SAS 9.4 package by cancer type [20]. ISCR’s variables included patient’s age, sex, race/ethnicity, comorbidities, primary payer, 2010 census tract and block group, residential rural urban community area (RUCA). A combined race ethnicity variable was created from the ISCR’s variables with the following categories: Non-Hispanic White, Non-Hispanic Black, Non-Hispanic Other/Unknown and Hispanic (any race). An Elixhauser comorbidity index software tool package utilizing the ISCR’s comorbidity and secondary diagnoses registry data produced a comorbidity index and grouped into three categories (0, 1 or 2 or more) for the number of comorbidities [21]. Patient primary payer insurance classifications were collapsed into six categories: 1) private insurance, 2) Medicaid, 3) Medicare, 4) uninsured or self-pay, 5) other insurance and 6) unknown. The RUCA was established at the level of the patient’s 2010 census tract, creating four categories: metropolitan, micropolitan, small town, and rural areas [22].

### Geospatial Visualization and Hotspot Determination

The analytical procedure adopted in this study for hotspot determination is described briefly here, with greater detail in previous publications [15, 23, 24]. The essential method we used in this study for quantifying the local disease intensity is called kernel ratio estimation (KRE) [15, 23]. The basic idea of KRE is to define a circular neighborhood (kernel) around a disease case location and compare the actual patients-to-population ratio with simulated (expected) ratios [15, 23, 24].

For the background population, we used LandScan^™^ USA, a gridded population dataset available through the Oak Ridge National Laboratory [25]. Since the LandScan data only has the population count for each raster cell, we applied the age information from the US Census block group to the LandScan^™^ data by assuming the population in a raster cell has the same age structure as the corresponding Census block group. Both the modified LandScan data and the patient cohort were stratified into three age groups (Online Resource 1) to mitigate the effects of confounding by age. The age groups reflect the United States Preventive Services Task Force cancer screening guidelines in place at the time of diagnosis [26].

The analytical procedure is briefly described here, and further details can be found in previous publications [15, 22, 23]. The kernel method requires point locations, but the cohort data from the ISCR was aggregated at the block group level. To take advantage of KRE, we disaggregated the block group-level patient counts to points using a restricted (to the associated block group) and controlled (by the background (LandScan) population) Monte Carlo (RCMC) process and then running KRE with a 250-person adaptive bandwidth on the disaggregated points [27]. Since this disaggregation is a randomizing process, to estimate the uncertainty in this process, we ran RCMC 10 times, considering the output of each represents the actual locations of the patients.

To estimate the statistical significance of the local disease rate calculated by KRE, we generated simulated (expected) patient locations through an unrestricted (not limited to the block group polygon) but controlled (by the statewide background population) Monte Carlo process (UCMC) and then ran the KRE on the simulated patient locations using the same adaptive bandwidth applied to the patient locations disaggregated by RCMC. The KRE process was run on each of the three age groups and then the resulting estimates from the age groups were integrated into an overall estimate following the principle of direct adjustment. The statistical significance of the result of each RCMC iteration (i.e., the actual patient-to-population ratio at each location), that RCMC result was compared to 199 results from the UCMC simulations to determine the p-value. A location (represented by a raster cell) is considered to be within a hotspot area if the mean of its 10 p-values plus 2 standard deviations was < 0.01.

To further characterize the hotspots, we compared the absolute size and distribution of the identified hotspots both by cancer site and outcome measure. The sizes of the hotspot areas were compared to the total area of the state, and the area of overlap between outcome measures for each cancer site as well as for same outcome measure for different cancer sites was also calculated. The areas were reported in absolute square kilometers and as a percentage of the total area of the state. We also attributed case and late-stage diagnosis case counts to the hotspot areas by apportioning the block group’s counts based on the population distribution. From this, we established rates of late-stage diagnosis in hotspot and non-hotspot locations. A continuous hotspot area (which may consist of multiple hotspot pixels) with fewer than 11 cases was suppressed for patient confidentiality and stability purposes [28].

## RESULTS

Our final analytic cohort included 117,305 patients diagnosed with breast (n = 51,623), colorectal (n = 28,160), and lung (n = 37,522) cancer in Indiana between 2010 and 2019 (Table 1). The number of block groups in which patients resided varied between breast (n = 4,759), colorectal (n = 4,684), and lung (n = 4,745) cancer. Patients with breast cancer had a younger average age at diagnosis (62.5 years), a higher proportion of non-Hispanic Black patients, fewer comorbidities, higher proportion of patients with private insurance coverage. Conversely, patients with lung cancer were more likely to be non-Hispanic White, have one or more comorbidities, and have Medicare insurance coverage.

**Figure 2:**
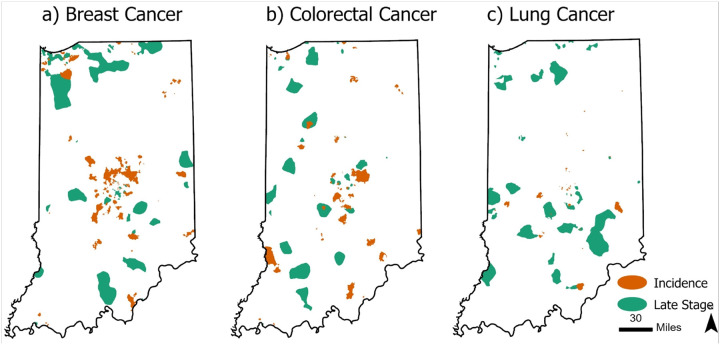
Geospatial hotspots of incidence and late-stage cancer in Indiana for a) breast, b) colorectal, and c) lung cancer, 2010–2019

Overall, we identified 69, 44, and 30 incidence hotspots and 33, 29, and 33 late-stage hotspots for breast, colorectal, and lung cancer, respectively (Fig. 2). The average proportion of late-stage breast cancer in these hotspots was 45.6% (95% CI 44.5–46.8) compared to 31.8% in non-hotspot areas of the states. For patients with colorectal cancer, the average proportion of late-stage cancer in hotspots was 57.0% (95% CI 54.2% – 59.8%) compared to 40.4% in non-hotspot areas of the states. In lung cancer, the average proportion of late-stage disease in these hotspots was 72.0% (95% CI 70.0–73.9%) compared to 58.0% in non-hotspot areas of the states.

The hotspots for breast cancer incidence totaled approximately 2,699 km^2^ and tended to be on the outskirts of major cities, including Indianapolis, Fort Wayne, Gary, and South Bend as well as in major college towns of Bloomington and Lafayette (Fig. 2a and Table 2). Conversely, hotspots of later-stage diagnoses tended to be closer to or within cities, including Indianapolis, South Bend, and Gary. Overlap with late-stage hotspots occurred in an area of only 128 km^2^ or approximately 4.7% of incidence hotspots for breast cancer (Table 2, Online Resource 2).

Incidence hotspots for colorectal cancer encompassed 2,078 km^2^, 2.2% of the total state area (Table 2; Online Resource 2). In evaluating hotspots for colorectal cancer, there appears to be closer proximity and more overlap between incidence and late-stage hotspots (Fig. 2b). Incidence and late-stage colon cancer hotspots occur both within and outside of metropolitan areas. Overlap with late-stage hotspots occurred in an area of 165 km^2^, only 7.9% of incidence hotspots for colorectal cancer (Table 2).

For lung cancer, we found fewer and smaller hotspots for both incidence and late-stage lung cancer (Fig. 2c). Furthermore, the area of the incidence hotspots for lung cancer totaled 396 km^2^, less than one percent of the total state area (0.4%). These results could be expected to occur by chance with the parameters of our Monte Carlo simulations (Table 3; Online Resource 2). Given that the total hotspot area is less than 1% of total state area, we can interpret these lung cancer incidence hotspots to have been “detected” by chance alone. However, the hotspots for late-stage lung cancer are statistically significant and included 5,671 km^2^, approximately 6.0% of the total state area, and were largely located in more rural areas (Fig. 2c and Table 3).

**Figure 3:**
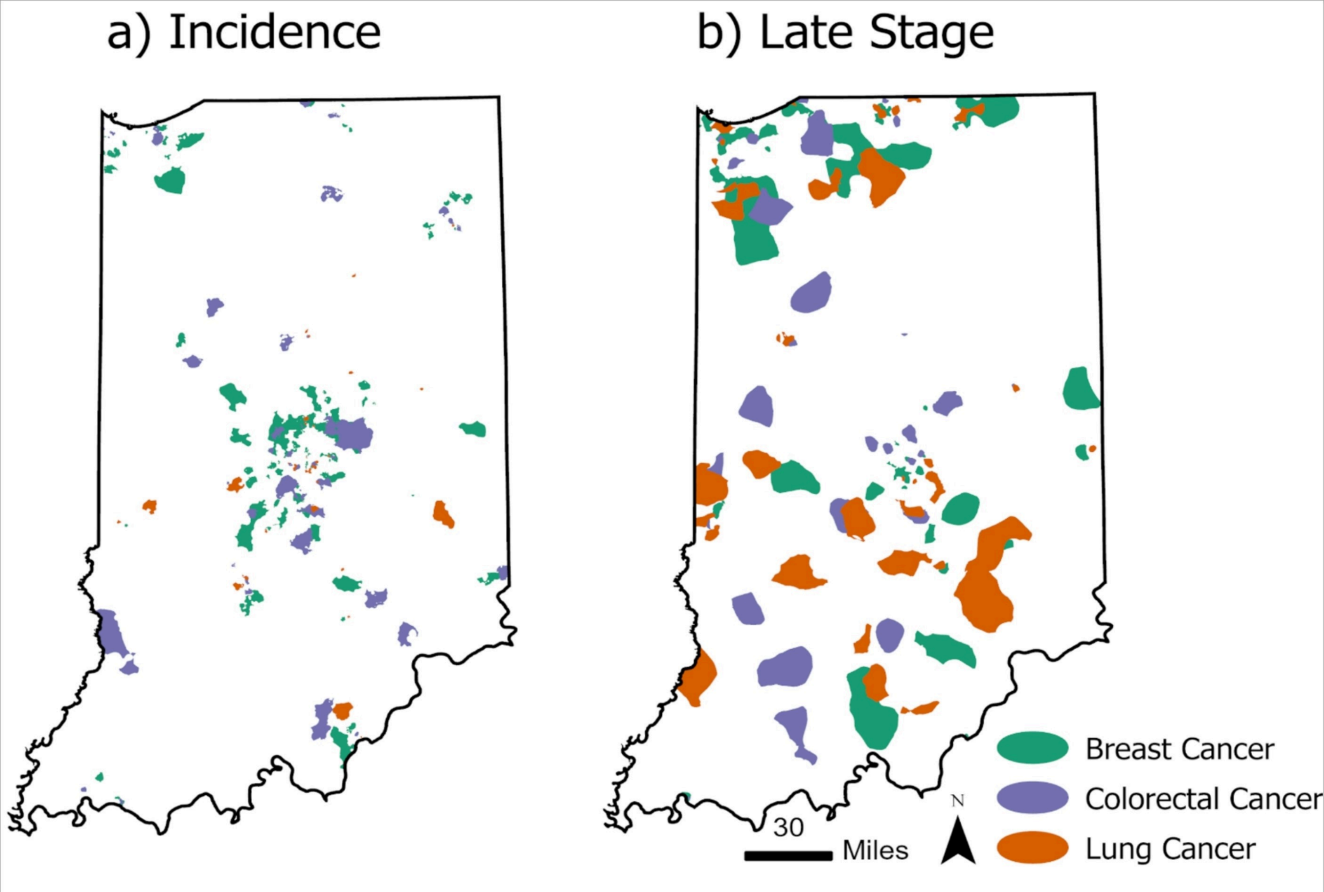
Geospatial Hotspots of a) incidence, b) late-stage diagnosis with breast, colorectal, and lung in Indiana, 2010–2019

The relationships of incidence and late-stage hotspots between the different cancer sites are presented in Fig. 3. The incidence hotspots for breast and colorectal cancer had the highest degree and area of overlap (428 km^2^ and 15.8% of hotspots overlap) and were in closer proximity to each other, especially in the center portion of the state (Fig. 3a and Table 3). There appears to be more isolated hotspots of colorectal and lung cancer, especially in the southern portion of the state. Shifting to comparing late-stage hotspots for the different cancer sites, late-stage breast and lung cancer hotspots had the greatest area and proportion of overlap (including 1,046 km^2^) followed by colorectal cancer and lung (499 km^2^) (Fig. 3b and Table 3). There is minimal overlap between all three cancer sites for both incidence and late-stage hotspots (6 km^2^ and 2 km^2^, respectively).

## DISCUSSION

In this study of 117,305 adult patients diagnosed with breast, colorectal, or lung cancer between 2010 and 2019 in Indiana, we demonstrated considerable heterogeneity in hotspots both between different outcome measures as well as between different cancer types. The proportional overlap between incidence and late-stage hotspots was greatest for lung cancer (8.0%) compared to either breast or colorectal cancer (4.7% and 7.9%, respectively). However, the overlapping area between incidence and late-stage hotspots totaled only 32 km^2^. The overlap for incidence hotspots between cancers was greater for breast and colorectal cancer hotspots (428 km^2^) and less between lung and either breast or colorectal cancer hotspots (16 km^2^ and 26 km^2^, respectively). The greatest degree in overlap between late-stage hotspots was between breast and lung cancer hotspots, with an area of 1,046 km^2^. Collectively, these results demonstrate marked variation both within and between cancer sites in localizing hotspots for two outcome measures along the cancer continuum.

Cancer control and prevention efforts rely on appropriate identification of populations at risk of developing or failing to receive treatment for cancer [8, 10, 29]. Traditional polygon-based geospatial methodology assumes uniform distribution of outcomes across large areas with considerable variation in population demographics, environmental exposures, and health care services [29–31]. Our use of disaggregated data and raster-based methodology demonstrated considerable geospatial heterogeneity of hotspots across the state of Indiana. These results reinforce the urgency to use place-based consideration in understanding drivers of and variation in cancer inequity across the continuum of disease [32].

The heterogeneity of hotspots seen in this study is most likely due to marked variation in the drivers of cancer development and cancer care delivery in general and in different cancer sites [33, 34]. Breast cancer incidence hotspots were located largely on the outskirts or edges of cities and in communities with the lowest degree of socioeconomic deprivation. This is consistent with prior work showing the inverse relationship between poverty and the incidence of breast and colorectal cancer [35]. Although with a relatively small area, there was more overlap between breast and colorectal incidence hotspots as compared to either site overlapping with lung cancer incidence hotspots. Conversely, lung cancer incidence is known to have more of a direct correlation with poverty [36]. As compared to breast or colorectal cancer, the vast majority of lung cancers are attributable to the patient-level behavior of smoking [37]. Our results demonstrated very few and a small area of lung cancer incidence hotspots in Indiana. Over this study period, Indiana was in the top third of states in terms of adult smoking rates and in the top 15% of states in terms of lung cancer incidence [38, 39]. As such, there may be more ubiquitous smoking rates across the state leading to lower probability of one area having disproportionately greater lung cancer risk than other (“hotspots”).

Compared to incidence hotspots, there was a greater area of late-stage hotspots and overlapping of late-stage hotspots between different cancer sites. These findings are likely related to shared drivers of late-stage diagnosis, especially for breast and colorectal cancer which have greater availability and accessibility of screening services [4, 11, 14]. Geographic and socioeconomic variation in access to care, including screening services, have been well documented for all breast, colorectal and lung cancer [4–6, 11, 13, 14, 29, 36, 37]. Indiana, similar to the rest of the country, has a relatively low screening rate of approximately 17% of high-risk patients [40]. We were therefore somewhat surprised by the large number and area of late-stage hotspots for lung cancer, expecting more homogenous distribution of late-stage diagnoses across the state. However, we suspect that similar barriers to accessing screening services result in the larger number and area of late-stage referrals. Interesting, the largest area of overlapping late-stage hotspots was between breast and colorectal cancer, suggesting a potential opportunity to coordinate screening services in these higher-risk patients and areas of the state [41].

These results have several different implications for both cancer control strategies and policy evaluation. First, these geospatial methods of disaggregating patient-location data to approximate more precise location based on underlying population density allow for more granular, discriminative mapping of higher risks areas while also maintaining patient confidentiality and data stability. There are marked variations in neighborhood environments, including racial and socioeconomic segregation, transportation resources, or carcinogen exposures, that occur at areas smaller than county or ZIP code. Such methods could improve cancer control measurement, evaluation, and planning for departments of health as well as informing targeted policies to address place-based drivers of adverse cancer outcomes. Given the highest overlap between late-stage breast and lung cancer hotspots, consideration should be given to potentially collocating screening interventions for both breast and lung cancer [42, 43]. However, given very little overlap of incidence hotspots, more cancer site-specific strategies should be employed to identify unique locations at higher risk of developing new cancers.

These results should be considered in the context of methodological limitations. First, the cancer registry data, although maintained with standardized variable definitions and reporting processes, are susceptible to coding errors that are often not immediately appreciated. Perhaps more relevant to the present study is that the ISCR lacks patient residential history which has significance, especially for incidence hotspots. Therefore, we are only able to map the locations of where patients lived at the time of diagnosis, without knowing the duration of residence or prior residencies. Since this methodology is an ongoing area of research and development, the current applications prevent determining the specific age-adjusted incidence rates or precise proportion of patients with late-stage cancer in each of these hotspots. However, our hotspots are similar in location (although more granular) compared to previously published cluster detection in Indiana, and we were able to estimate the proportion of hotspot’s incidence and late-stage cancer. Additionally, we are unable to discern additional geospatial attributes of these hotspots as many demographic or environmental data are available only at larger aggregated areas, such as census tracts, ZIP codes, or county. As such, these methods provide more geographic granularity in describing the location of hotspots, but are unable to further describe characteristics of the populations within hotspots. Finally, our findings may be state-specific and may not be generalizable outside of Indiana. The increasing recognition of both spatial and aspatial drivers of care will require more population-specific measurements and evaluations.

## CONCLUSION

In this study of over 117,000 patients in Indiana diagnosed with breast, colorectal, or lung cancer between 2010 and 2019, we found considerable spatial deviation of incidence and late-stage hotspots both within and between sites of disease. Within each cancer site, there was little overlap between incidence and late-stage hotspots, suggesting different cancer control measures for prevention and early detection within cancer sites. Incidence hotspots for breast and colorectal cancer were most likely to overlap while late-stage hotspots overlapped the most between breast and lung cancer, raising the potential for geographically collocating strategies for prevention of breast and colorectal cancer and earlier diagnosis of breast and lung cancer. These results demonstrate the critical importance of including spatial data while identifying disparities in cancer development and access to timely diagnosis.

## Supplementary Files

This is a list of supplementary files associated with this preprint. Click to download.

- GeospatialheterogeneityofcancerhotspotsOnlineResource1.docx
- GeospatialheterogeneityofcancerhotspotsOnlineResource2.docx

## Figures and Tables

**Table 1:** Indiana cohort demographic and clinical characteristics among those with breast, colorectal and lung cancer (N = 117,305) and diagnosed between 1/1/2010 and 12/31/2019.

	Breast (N = 51,623)	Colorectal (N = 28,160)	Lung (N = 37,522)
**Number of block groups (N = 14,188)**	4,759	4,684	4,745
**Age mean (std)**	62.5 (13.3)	67.3 (13.7)	69.2 (11.1)
**Female**	51,623 (100.0)	13,635 (48.4)	17,423 (46.4)
**Race/Ethnicity**			
Non-Hispanic White	45,418 (88.0)	24,977 (88.7)	34,169 (91.1)
Non-Hispanic Black	4,321 (8.4)	2,125 (7.6)	2,706 (7.2)
Non-Hispanic Other/Unknown	686 (1.3)	449(1.6)	252 (0.7)
Hispanic (Any Race)	1,196 (2.3)	609 (2.2)	395 (1.1)
**Number of Comorbidities**			
0	39,326 (76.2)	16,037 (57.0)	19,573 (52.2)
1	5,874 (11.4)	5,349 (19.0)	8,305 (22.1)
2+	2,389 (4.6)	3,572 (12.7)	5,995 (16.0)
Unknown	4,034 (7.8)	3,202 (11.4)	3,649 (9.7)
**Insurance Coverage**			
Private	18,830 (36.5)	6,894 (24.5)	6,027 (16.1)
Medicaid	2,664 (5.2)	1,368 (4.9)	2,228 (5.9)
Medicare	22,420 (43.4)	15,676 (55.7)	24,011 (64.0)
Uninsured/Self-Pay	990 (1.9)	732 (2.6)	1,008 (2.7)
Other	5,419 (10.5)	2,346 (8.3)	2,278 (6.1)
Unknown	1,300 (2.5)	1,144 (4.1)	1,970 (5.3)
**Rural Urban Commuting Area**			
Metropolitan	40,249 (78.0)	20,665 (73.4)	27,838 (74.2)
Micropolitan	6,911 (13.4)	4,501 (16.0)	5,973 (15.9)
Small Town	2,951 (5.7)	1,982 (7.0)	2,480 (6.6)
Rural	1,512 (2.9)	1,012 (3.6)	1,231 (3.3)
**Stage at Diagnosis**			
0	8,391 (16.3)	1,361 (4.8)	108 (0.3)
1	22,421 (43.4)	5,492 (19.5)	8,470 (22.6)
2	11,434 (22.2)	5,822 (20.7)	3,267 (8.7)
3	3,649 (7.1)	6,437 (22.9)	7,202 (19.2)
4	2,422 (4.7)	5,204 (18.5)	14,976 (39.9)
Unknown, Occult	3,306 (6.4)	3,844 (13.7)	3,499 (9.3)
**Late-Stage Diagnosis**			
No	31,039 (60.1)	12,675 (45.0)	11,845 (31.6)
Yes	17,278 (33.5)	11,641 (41.3)	22,178 (59.1)
Unknown	3,306 (6.4)	3,844 (13.7)	3,499 (9.3)

**Table 2:** Area for individual hotspots and the overlap of hotspots within and between cancer sites.

	Primary Hotspots (square kilometers)			Overlap of Hotspots Between Cancer Sites (square kilometers)			
	Breast	Colorectal	Lung	Breast - Colorectal	Breast-Lung	Colorectal-Lung	Breast-Colorectal-Lung
**Incidence Hotspots**	**2,699**	**2,078**	**396**	428	16	26	6
**Late-Stage Hotspots**	**6,836**	**4,132**	**5,671**	408	1,046	499	2
**Overlap of Hotspots** (square kilometers)	128	165	32				

## Data Availability

This study used data available under review and data use agreement with the Indiana State Cancer registry. Request to access data will be directed to the registry at: https://www.in.gov/health/cdpc/cancer/cancer-registry/
